# Supplementary material for: The Influence of Magnetic Field and Nanoparticle Concentration on the Thin Film Colloidal Deposition Process of Magnetic Nanoparticles: The Search for High-Efficiency Hematite Photoanodes
Source: Nanomaterials (Basel). 2022 May 11;12(10):1636. doi: 10.3390/nano12101636 (PMC9146261; doi:10.3390/nano12101636)
Supplement: Supplementary file 1 [file nanomaterials-12-01636-s001.zip › nanomaterials-1687218-supplementary.pdf]

nanomaterials

Supporting Information

# The Influence of Magnetic Field and Nanoparticle Concentration on the Thin Film Colloidal Deposition Process of Magnetic Nanoparticles: The Search for High-Efficiency Hematite Photoanodes

**Murillo Henrique de Matos Rodrigues [1,2], Joao Batista Souza Junior [2] and Edson R. Leite [1,2,*]**

1 Department of Chemistry, Federal University of São Carlos, Via Washington Luiz, km 235, São Carlos, SP, 13565-905, Brazil; murillo.matos@lnnano.cnpem.br
2 Brazilian Nanotechnology National Laboratory (LNNano), Brazilian Center for Research in Energy and Materials (CNPEM), Campinas, SP 13083-970, Brazil; joao.junior@lnnano.cnpem.br
* Correspondence: edson.leite@lnnano.cnpem.br

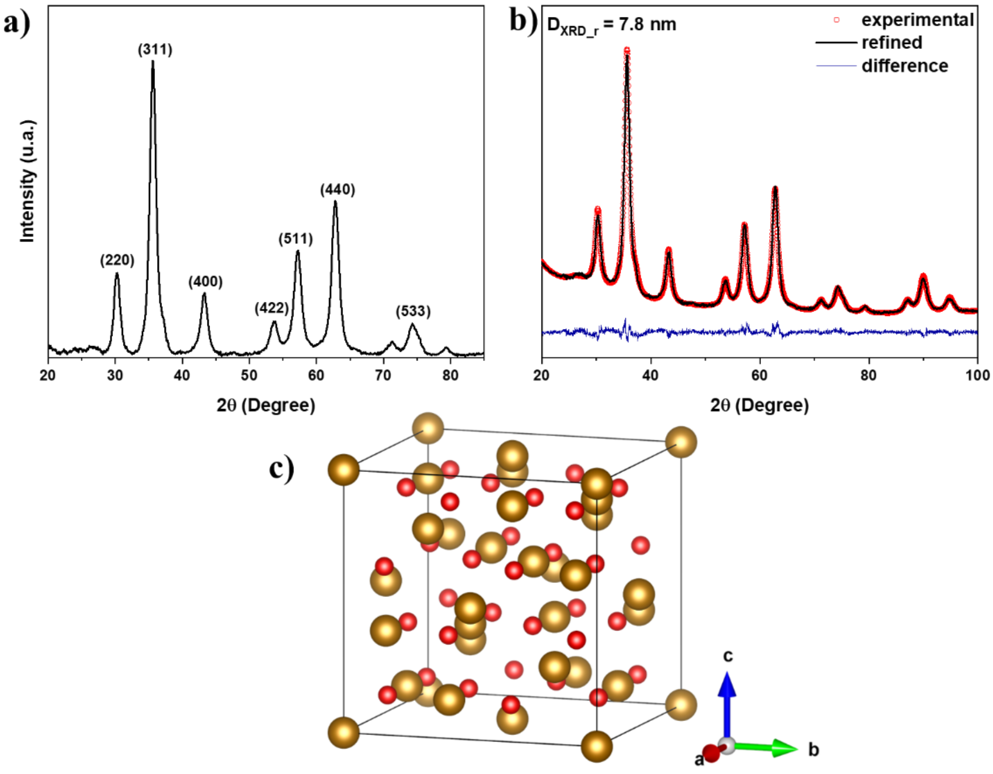


**Figure S1.** X-ray diffraction patterns of magnetite nanoparticles: (**a**) experimental; (**b**) Rietveld refinement; and (**c**) unit cell of magnetite.

**Table S1.** Rietveld size, lattice parameter, Rwp extracted from simulated data, and R-Bragg.

| | Rietveld Size (nm) | Lattice Parameter (Å) | Rwp (%) | R-Bragg |
|---|---|---|---|---|
| Magnetite Nanoparticles | 7.8 | 8.378 | 8.54 | 0.956 |

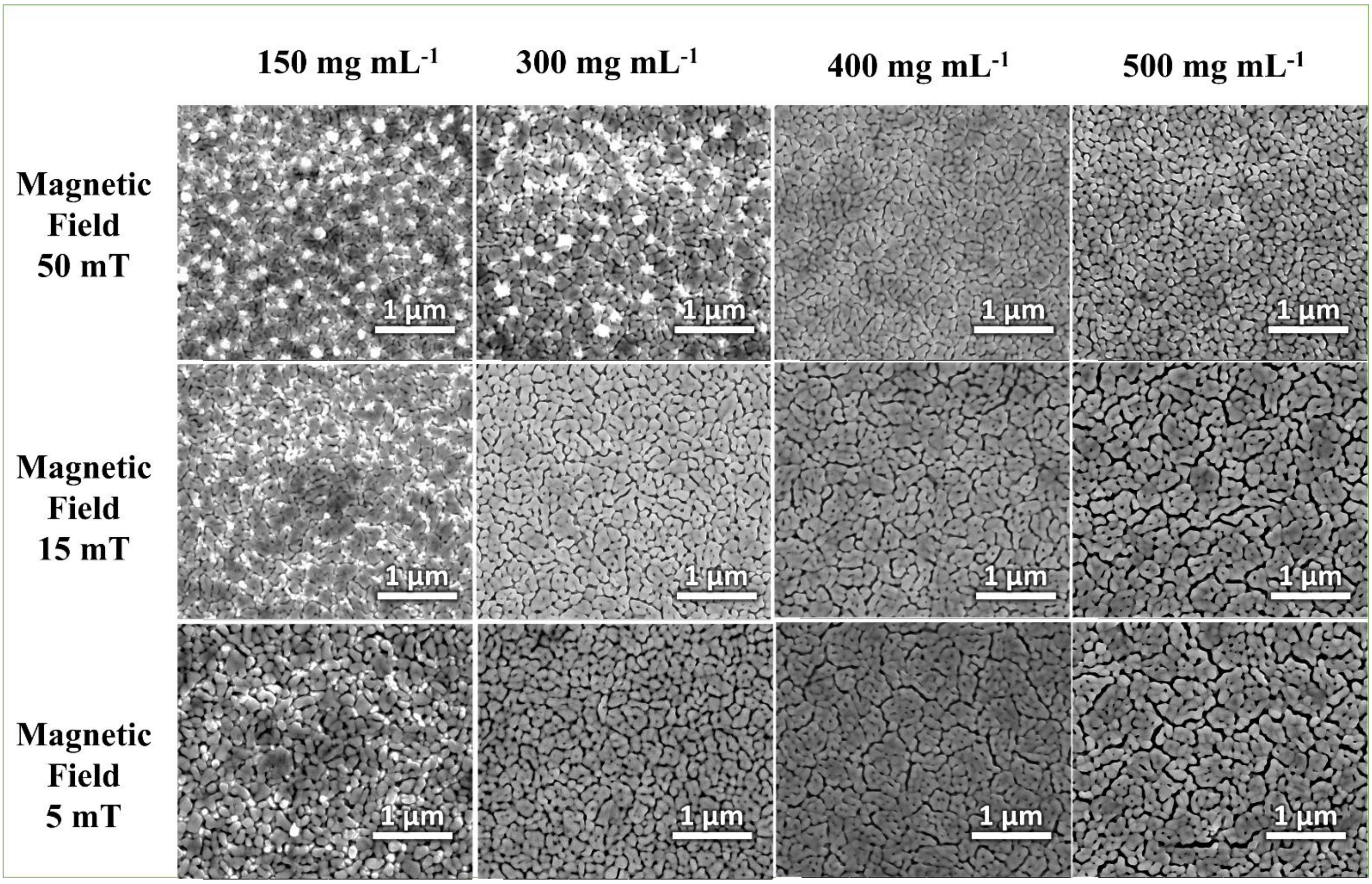


**Figure S2.** SEM images (top-view) of hematite nanostructures obtained with different magnetic fields and nanoparticles concentrations.

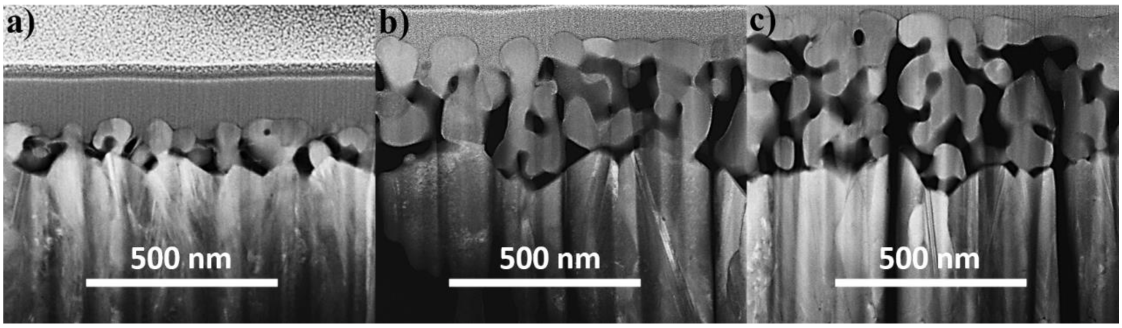


**Figure S3.** SEM images cross section of hematite nanostructures obtained with 500 mg mL$^{-1}$ at: (a) 50 mT magnetic field; (**b**) 15 mT magnetic field; and (**c**) 5 mT magnetic field.

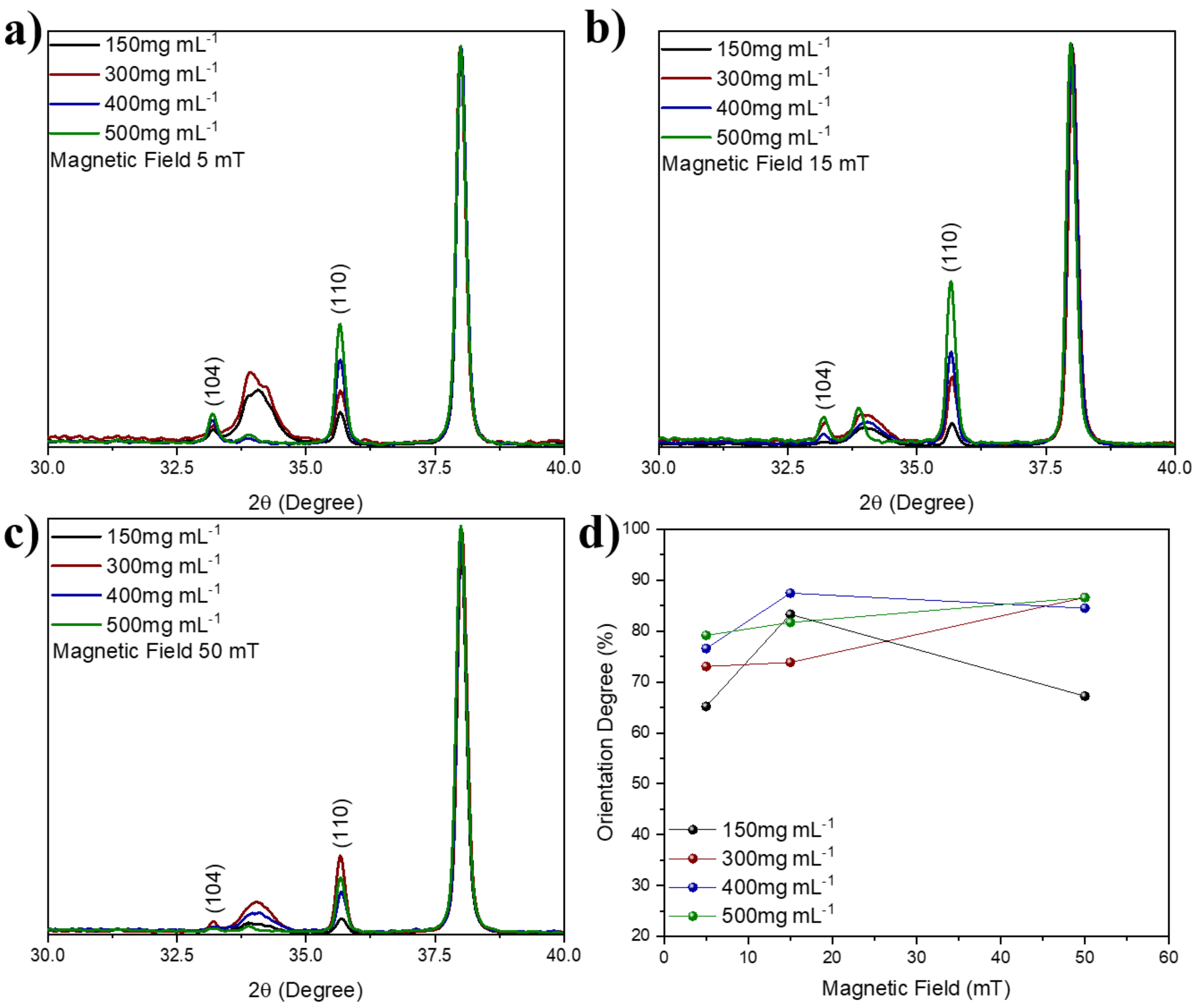


**Figure S4.** X-ray diffraction peaks of hematite thin films obtained at: (**a**) 5 mT magnetic field; (**b**) 15 mT magnetic field; (**c**) 50 mT magnetic field; and (**d**) percentage of film orientation along the 110 plane as a function of the magnetic field.

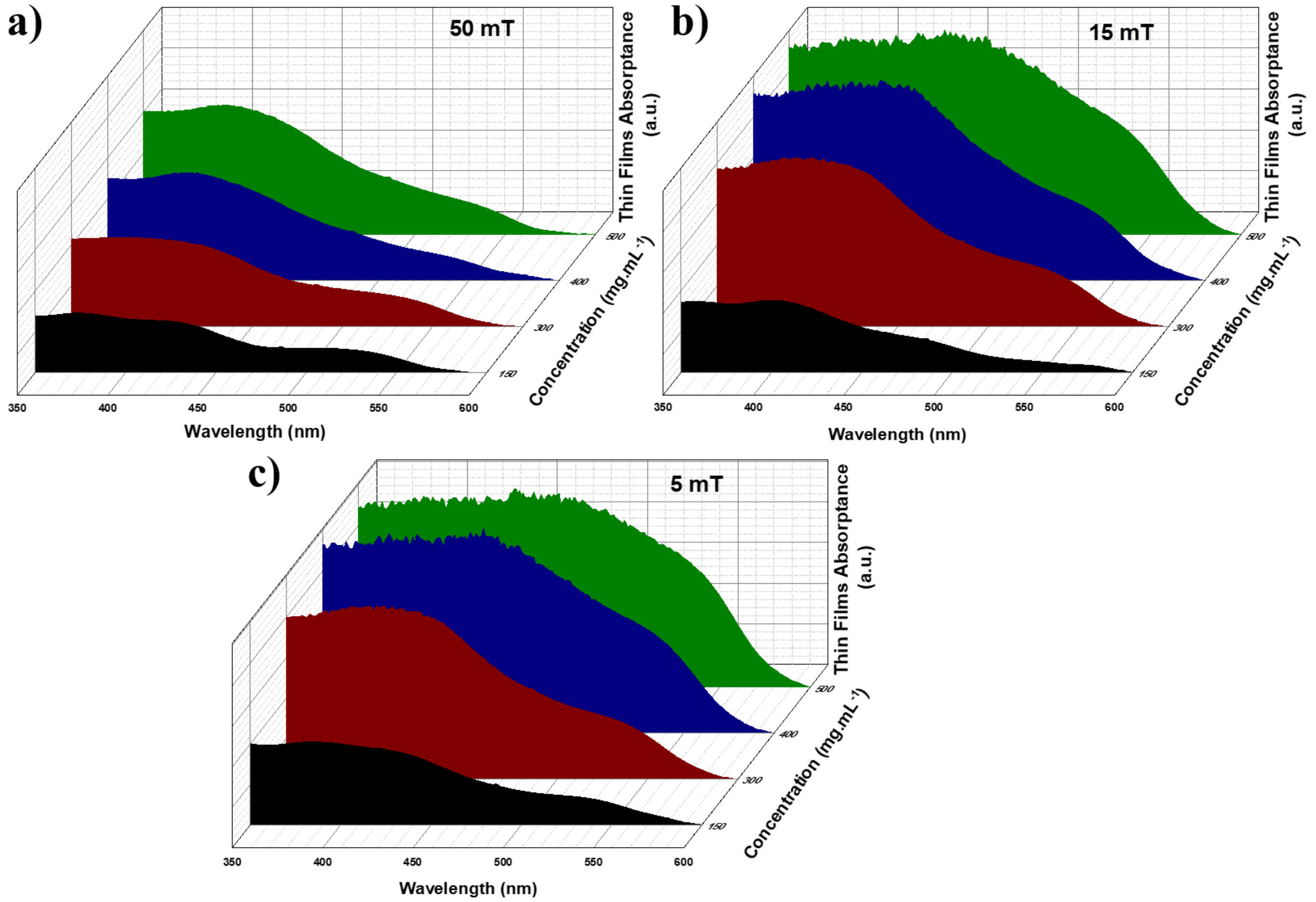


**Figure S5.** UV-vis spectra of the hematite films prepared with different nanoparticles concentration at: (**a**) 5 mT magnetic field; (**b**) 15 mT magnetic field; and (**c**) 50 mT magnetic field.

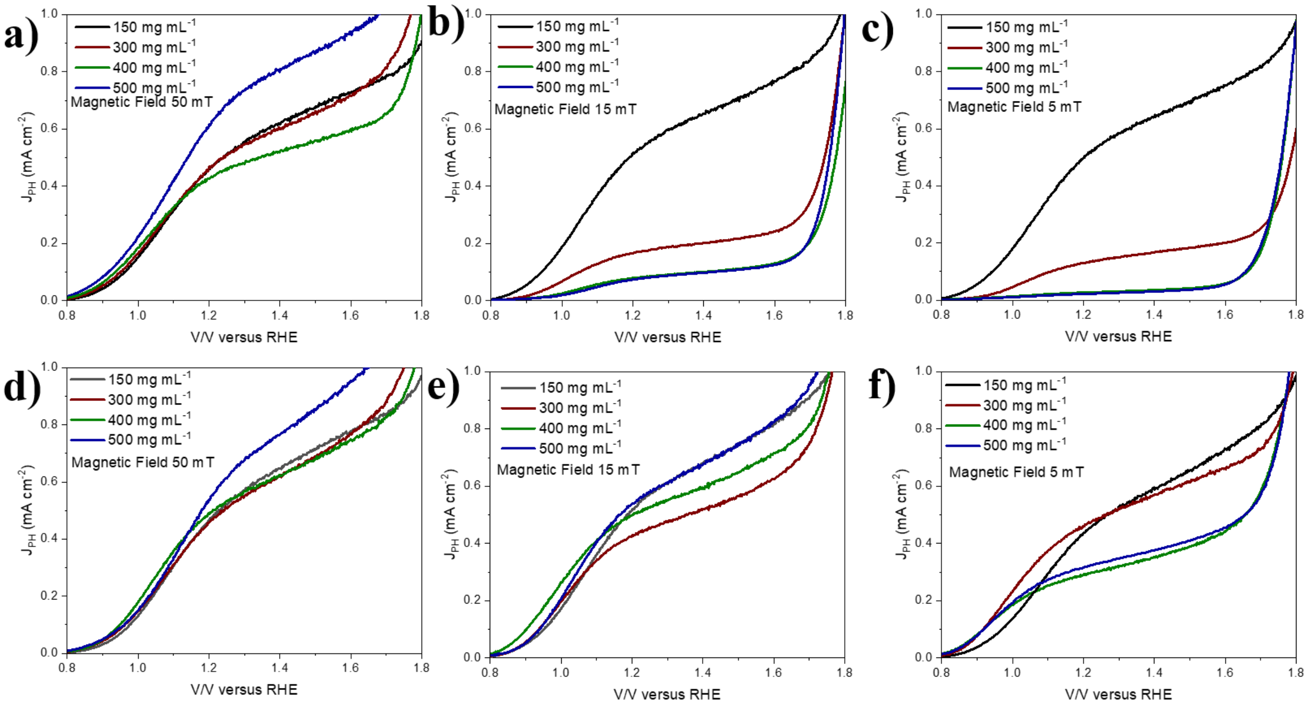


**Figure S6.** Current-potential curves of hematite thin films obtained with different nanoparticles concentration at: (**a**) 5 mT magnetic field under front illumination; (**b**) 15 mT magnetic field under front illumination; (**c**) 50 mT magnetic field under front illumination; (**d**) 5 mT magnetic field under back illumination; (**e**) 15 mT magnetic field under back illumination; and (**f**) 50 mT magnetic field under back illumination.

**Table S2.** Parameters of two-teta (2Θ), full width at half maximum (FWHM), and crystallite size of hematite thin films obtained with different concentration ate 5, 15 and 50 mT magnetic field.

| | **150 mg mL⁻¹** | | | **300 mg mL⁻¹** | | | **400 mg mL⁻¹** | | | **500 mg mL⁻¹** | | |
|---|---|---|---|---|---|---|---|---|---|---|---|---|
| | **2Θ (°)** | **FWHM** | **Crystallite Size (nm)** | **2Θ (°)** | **FWHM** | **Crystallite Size (nm)** | **2Θ (°)** | **FWHM** | **Crystallite Size (nm)** | **2Θ (°)** | **FWHM** | **Crystallite size (nm)** |
| **5 mT** | 35.668 | 0.191 | 43.7 | 35.675 | 0.222 | 37.6 | 35.664 | 0.204 | 40.9 | 35.666 | 0.199 | 42.0 |
| **15mT** | 35.686 | 0.200 | 41.8 | 35.690 | 0.205 | 40.7 | 35.667 | 0.193 | 43.3 | 35.667 | 0.193 | 43.3 |
| **50mT** | 35.688 | 0.214 | 38.9 | 35.674 | 0.200 | 42.4 | 35.692 | 0.202 | 41.3 | 35.679 | 0.199 | 42.0 |
